# Computational tools to study RNA-protein complexes

**DOI:** 10.3389/fmolb.2022.954926

**Published:** 2022-10-07

**Authors:** Sneha Bheemireddy, Sankaran Sandhya, Narayanaswamy Srinivasan, Ramanathan Sowdhamini

**Affiliations:** ^1^ Molecular Biophysics Unit, Indian Institute of Science, Bangalore, India; ^2^ Department of Biotechnology, Faculty of Life and Allied Health Sciences, M.S. Ramaiah University of Applied Sciences, Bengaluru, India; ^3^ National Centre for Biological Sciences, TIFR, GKVK Campus, Bangalore, India; ^4^ Institute of Bioinformatics and Applied Biotechnology, Bangalore, India

**Keywords:** computational prediction, RNA-protein complex, RNA-protein interaction, RPI, interface prediction, machine learning, deep learning

## Abstract

RNA is the key player in many cellular processes such as signal transduction, replication, transport, cell division, transcription, and translation. These diverse functions are accomplished through interactions of RNA with proteins. However, protein–RNA interactions are still poorly derstood in contrast to protein–protein and protein–DNA interactions. This knowledge gap can be attributed to the limited availability of protein-RNA structures along with the experimental difficulties in studying these complexes. Recent progress in computational resources has expanded the number of tools available for studying protein-RNA interactions at various molecular levels. These include tools for predicting interacting residues from primary sequences, modelling of protein-RNA complexes, predicting hotspots in these complexes and insights into derstanding in the dynamics of their interactions. Each of these tools has its strengths and limitations, which makes it significant to select an optimal approach for the question of interest. Here we present a mini review of computational tools to study different aspects of protein-RNA interactions, with focus on overall application, development of the field and the future perspectives.

## 1 Introduction

The roles of RNA, as coding and non-coding RNA in transcription, translation, gene regulation, transport, catalysis, cell division and many other processes continues to expand with advancement in methods to study them (Crick, 1970; Pyle, 1993; Moore and Steitz, 2002; Sugiura et al., 2011; Barbieri and Kouzarides, 2020; Christopoulou and Granneman, 2022; Perez et al., 2022; Song et al., 2022). The growing repertoire of non-coding RNA types (ncRNA: ribosomal RNA, transfer RNA, miRNA, snRNA/small nuclear RNA, piRNA, catalytic RNA, small nucleolar RNA, *etc.*) shows their tremendous diversity in sequence, structure, subcellular localization, and function. Central to their role in fundamental biological processes are RNA-protein interactions (RPI) that primarily involve modular RNA-binding proteins (RBP), although exceptions exist (Castello et al., 2012). Typically, this recognition is effected through an RNA-binding domain (RBD) such as RNA recognition motif (RRM), hnRNP K homology (KH) domain, DEAD box helicase domain (DDX), pumilio homology domain (PUM-HD) *etc.* and may involve recognition of specific sequence motifs on RNA or such sequences in specific structural contexts (Jolma et al., 2020). Alternatively, this recognition is based on structures adopted by RNA such as in G-quadruplexes (Kharel et al., 2020). RBPs are also known to bind RNA through intrinsically disordered regions (IDRs), resulting in extended interaction interfaces and higher order assemblies (Calabretta and Richard, 2015). More recently, RNA interactome captures (Gerstberger et al., 2014; Hentze et al., 2018) have revealed that up to 10% of the entire proteome may be bound to RNA emphasizing their importance in function. Indeed, such studies are contributing to excellent resources for cataloguing the complete set of protein-RNA interactions (RPI) across various cell types and whole organisms (Baltz et al., 2012; Castello et al., 2012; Kwon et al., 2013; Mitchell et al., 2013; Beckmann et al., 2015; Matia-González et al., 2015; Liepelt et al., 2016; Sysoev et al., 2016; Wessels et al., 2016; Hentze et al., 2018; Trendel et al., 2019; Urdaneta et al., 2019). Perturbations of RNA-RBP interactions are known to result in cellular dysfunction and have been implicated in many diseases (Allerson et al., 1999; Batista and Chang, 2013). It is, therefore, important to characterize RNA-proteins interactions.

Several experimental approaches are available to study the physical association between individual proteins and RNA molecules and these have been described in excellent reviews (Darnell, 2010; Ramanathan et al., 2019; Licatalosi et al., 2020; Cozzolino et al., 2021; Gräwe et al., 2021). Broadly, these approaches are grouped into RNA-centric or protein-centric approaches. While the former attempts to study proteins that associate with an entire population of RNA or an RNA that is expressed in a specific cell type or tissue (Campbell and Wickens, 2015; Cook et al., 2015; Gerber, 2021), the latter aims to pull down all RNA that specifically interacts with a protein. These methods may involve antibody-based immunoprecipitation of RBP and interacting RNA or involve crosslinking between protein and RNA (Ramanathan et al., 2019). RNA associated with the protein is then isolated and further analysed by microarray, sequencing, hybridization or polymerase chain reaction-based methods. Here, not only can the actual RNA sequence be determined, but also its abundance in the immunoprecipitated sample can be estimated and this is useful to map the binding site of the RBP of interest to the RNA molecule (Gagliardi and Matarazzo, 2016). A definitive way to identify RNA-binding residues or study RNA-protein interfaces (i.e., amino acid residues that directly contact RNA) is to extract them from a high-resolution experimental structure of a protein–RNA complex. However, structures of more than 80% of the protein-RNA complexes are known (Bienert et al., 2017; Dimitrova-Paternoga et al., 2020) owing to challenges in biophysical techniques such as complicated measurement process, time, resolution limits, cost-intensive steps *etc.* (Ke and Doudna, 2004; Scott et al., 2008; Pai et al., 2017). Added challenges in the determination of structures of protein-RNA complexes include the recognition of RNA sequences of optimal size that will bind the protein specifically and stably and the inherent flexibility of such regions which can affect structural stability and structure determination efforts. The inherent diversity of RNA structures and proteins and the presence of intrinsically disordered regions presents an enormous challenge to the area as well (Dyson, 2012; Ottoz and Berchowitz, 2020; Vandelli et al., 2022). The cost and effort to experimentally capture and measure strengths of all biologically important RNA–protein complexes is challenging due to these ique complexities. Although many experimental methods are now available to characterize protein-RNA interactions, mechanistic details of these reactions are still known. However, the availability of protein–RNA complex data from diverse experiments serve as ideal candidates for computational data analysis that can develop knowledge-based trends and rules to characterize such interactions. Indeed, several reliable computational methods that have derived patterns from the analysis of large ensembles of data have gained traction in predicting protein–RNA interaction (RPI) sites. Such methods when complemented with experimental data have been useful to analyze large datasets, to generate various hypothesis on interactions that can be tested again through experiment (Jiang et al., 2020; Teimouri and Maali, 2020; Zhang et al., 2021a). Some of the promising outcomes include prediction of the potential RNA binding sites in SARS-CoV-2 nucleocapsid protein revealing potential drug targeting sites (Cubuk et al., 2021), or a more detailed derstanding of the catalytic core formation through studies of spliceosomes and the role of allostery in mRNA interaction with ribosomes (Bao et al., 2018; Bheemireddy et al., 2021).

In this mini review, we provide an overview of existing computational methods to accurately predict RPI and analyze such interfaces. Our review broadly categorises these tools into sequence-based predictions, that tap into features in protein or RNA sequence, and structure-based computational methods that derive from available crystal structures of protein-RNA complexes and enable prediction of protein-RNA interactions. We have broadly classified these approaches based on their application and relevance in specific analysis and briefly describe and highlight well cited methods in each sub-section. These subsections are grouped based on the nature of input data used for the various analyses and enable a new user to choose an appropriate tool, that is relevant to their specific application interests.

## 2 Computational methods to predict protein-RNA interactions

**FIGURE 1:**
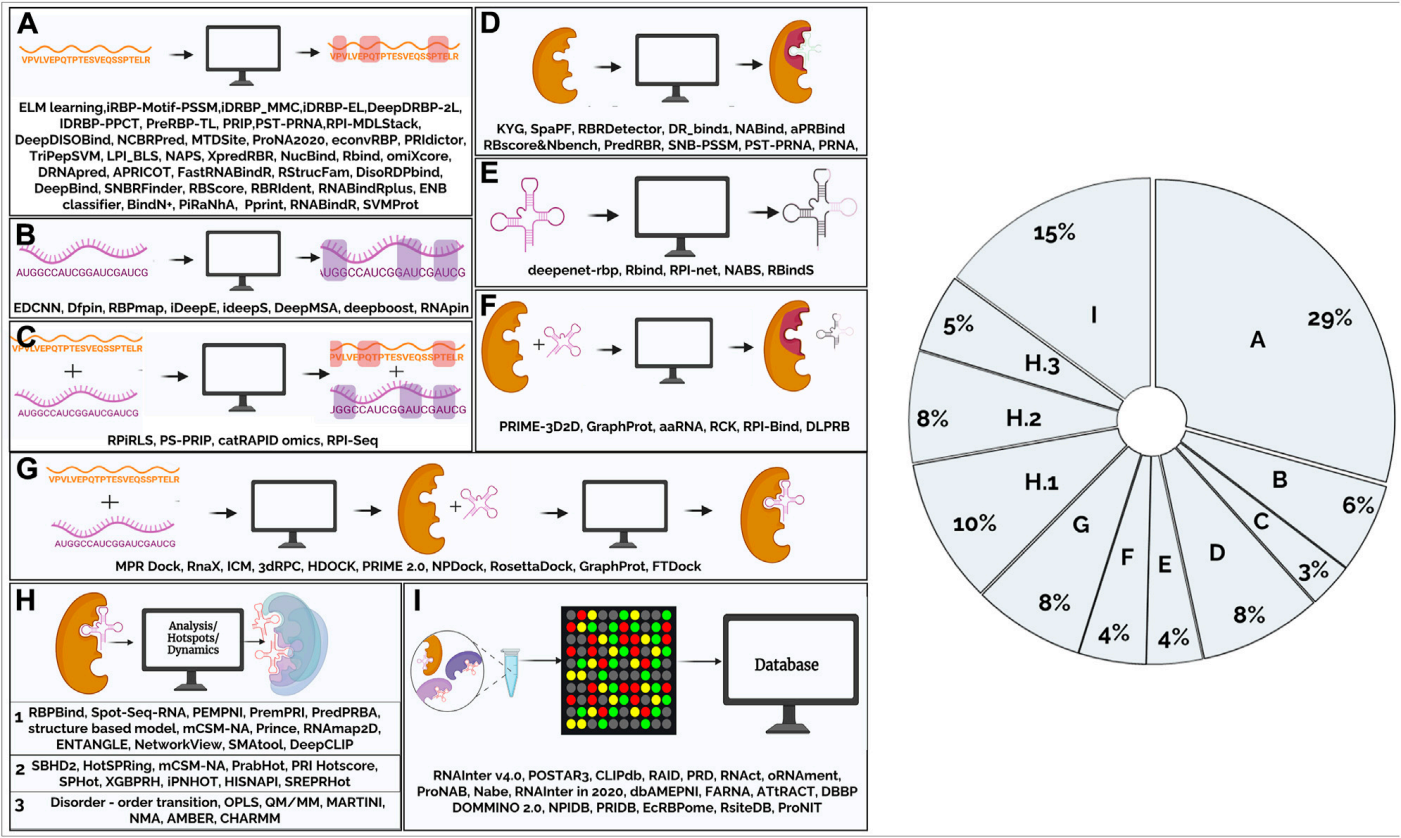
Figure summarizes the tools that are available to study protein-RNA interactions that have been classified based on their function. **(A)**. Computational tools for predicting amino acid residues in protein-RNA interactions using protein sequence (2.1.1) **(B)**. Computational tools for predicting nucleotides in protein-RNA interactions using RNA sequence (2.1.2) **(C)**. Computational tools for predicting amino acid residues and nucleotides in protein-RNA interactions using sequence information. (2.1.3) **(D)**. Computational tools for predicting amino acid residues in protein-RNA interactions using protein structural information (2.2.1) **(E)**. Computational tools for predicting nucleotides in protein-RNA interactions using RNA structural information (2.2.2) **(F)**. Computational tools for predicting amino acid residues and nucleotides in protein-RNA interactions using protein, RNA structural and sequence information (2.2.3) **(G)**. Computational tools for modelling/docking of protein-RNA complexes (2.3) **(H)**.1. Computational tools for analysis of protein-RNA complexes (2.4) **(H)**.2 Computational tools for prediction of hotspots in protein-RNA complexes (2.5) **(H)**.3. Computational tools to study dynamics using RNA-protein complex structure as input (2.6) **(I)**. Databases (2.7). Pie-chart shows main areas in RNA-protein interaction methods. From the figure, it is evident that majority of the tools were developed for prediction of amino acid residues and very few tools for modelling protein-RNA interactions. Nearly, 30% of the tools are for predicting interactions in the protein-RNA complexes given a protein query sequence.

Over the past decade, computational methods to predict RPI have been developed using either protein/ RNA sequence features (such as residue identity or physicochemical properties) and structure-based methods that use structure-derived features (such as solvent-accessible surface area or secondary structure) to make predictions. Walia et al., have reviewed tools for sequence-based methods (Walia et al., 2017) while structure-based methods that use secondary and tertiary structure of RNA have been described elsewhere (Zhang et al., 2015). With the availability of diverse experimental data there is tremendous interest and increase in development of methods that can learn trends from the data and arrive at predictive models. Computational tools to predict RPI vary in complexity and are based on either a combination of a few features or involve the application of network-based approaches/ machine learning (ML)-based methods such as deep learning, that capture hierarchical representations of intrinsically hidden data features. Collectively, these independent approaches have significantly prompted the development of a variety of tools that (Figure 1) can be classified based on their applications and aspects of RPI that they predict. In the following sections, we have grouped these tools into broad functional categories based on the input data and present the major tools to computationally infer RPI (Supplementary Table S1).

### 2.1 Methods that predict protein-RNA interactions using sequence information

#### 2.1.1 Protein sequence as input

Available experimental datasets enable the mapping of sequence motifs to binding scores. This, in turn, results in large volumes of such labelled and training data from diverse experiments and acts as an appropriate input for deep learning methods such as Alipanahi et al., 2015, that can derive patterns and build predictive models based on the training dataset. The input data used for training are obtained from various high-throughput experiments such as protein-binding microarrays, RNAcompete assays, ChIP-seq and HT-SELEX. The method has been shown to efficiently predict binding specificities of *in vivo* data and to identify deleterious genomic variants. With over 2000 citations, this scalable and modular pattern discovery method is a popular application of ML-based learning methods with important applications in predicting protein-RNA interactions (Supplementary Table S1). PPrint (Kumar et al., 2008) is another notable application that uses support vector machines to predict such interactions. Prediction models in these studies were trained on RNA-binding protein chains that were extracted from available crystal structures of RNA-protein complexes. An added advantage is the inclusion of evolutionary information through position-specific scoring matrices (PSSMs) derived for the sequence homologues of the training dataset. Other platforms, such as RStrucfam (Ghosh et al., 2016) use Hidden Markov Models (HMM) of RNA binding protein families (derived from sequence and structure databases) to recognize such properties of a protein query starting from mere sequence information. Another popular method employs convolutional neural networks and an extreme learning machine (ELM) classifier, to extract features from RNA and protein interaction datasets that are obtained from structures of known complexes of proteins and ncRNA. Representations of such datasets through numerical matrices and application of a learning classifier have been shown to effectively predict RPI in model organisms (Wang et al., 2020).

#### 2.1.2 RNA sequence as input

Just as the sequence features of proteins in RBP have contributed to the development of several methods to recognize such signatures in proteins, several methods are available to predict regions involved in such interactions in RNA as well. These motifs have been identified as targets of RBPs through experimental methods such as immunoprecipitation or cross-linking methods. Several tools have been developed to recognize RNA motifs/ sequences that bind proteins (Supplementary Table S1) by determining the short RNA sequence motifs that are known to occur at the interface of known protein-RNA complexes. Approaches such as RBPmap (Paz et al., 2014) allow the users to select motifs from a database of experimentally characterized motifs, that have been extracted from literature as a PSSM and use a Weighted-Rank (WR) approach to predict binding sites in query RNA sequences. A match score is computed for the motif per each position in the sequence in overlapping windows and its significance is evaluated taking into consideration the genome of interest. Properties of the motif environment, including the clustering propensity of binding sites and the overall tendency of such regions to be conserved provide additional advantages. Further, background model scores are employed to capture the significance of a match in the context that they occur in, in splice sites, 5′ and 3’ UTRs, non-coding RNAs and mid-intron/intergenic regions. RNApin (Panwar and Raghava, 2015) predicts such interaction sites using the trinucleotide composition profile of RNA and features extracted from RNA sequences using support vector machine (SVM). The method achieves accurate prediction and mapping of RBP binding sites on any input RNA sequence(s), provided by the users. While developed for organisms such as *Drosophila*, human and mouse, predictions can be made for other organisms as well.

The amount of experimentally verified RBP binding sites using CLIP-seq has exploded in recent times. Although variable between experiments, these data can serve as training set for machine learning models to predict missing RBP binding sites that may not be detected in some experiments. Methods such as Deepboost (Li et al., 2017) use a machine learning approach, called DeBooster, to accurately model the binding sequence preferences and identify the corresponding binding targets of RBPs from CLIP-seq data. ideepE (Pan and Shen, 2018) and ideepS (Pan et al., 2018) use a convolutional neural network-based approach that is trained on experimentally verified binding motifs from CLIP-seq data to identify RBP binding nucelotides. Here, known motifs were split into fixed length subsequences or padded into fixed length groups. These were employed to train convolutional neural networks (CNNs), to learn and extract high-level features that can identify sequence and structure binding motifs. Such methods were shown to achieve high accuracy to the order of 0.85.

Methods such as DFPin (Zhao et al., 2022) use the cascade structure of deep forest methods to extract key features based on RNA mono-nucleotide composition. Others such as EDCNN (Wang et al., 2022) address the issues of high-dimensionality, data sparsity and low model performance by combining evolutionary algorithms and different gradient descent models to optimize RNA-binding predictions. Integrated web servers such as RBPsuite (Pan et al., 2020) make use of deep learning, to predict nucleotides involved in protein-RNA interactions both in linear and circular RNA. Updates in the programs or training data are addressed at the server end. Such servers are not only useful to expand our knowledge of RBP-binding RNAs, but also, to investigate the effect of mutations on binding RBPs in RNA sequences, since they provide a binding score for a predicted interaction. Typically, these tools achieve an accuracy of 0.7–0.85.

#### 2.1.3 Protein and RNA sequence as input

Apart from the above-mentioned tools, there are few tools which consider both amino-acid and RNA sequence information, to predict residues involved in RNA-protein interactions. catRAPIDomics (Armaos et al., 2021), which can be used for large scale data analysis, uses a HMM-based algorithm to combine secondary structure, hydrogen bonding and van der Waals contributions and predicts protein-RNA associations with great accuracy. RPISeq (Muppirala et al., 2011) is another method that employs machine learning classifiers for predicting such interactions using sequence information. Here, in addition to predicting if a protein and RNA interact, added functionalities of the web-based application include predictions of interactions between a protein sequence and up to 100 RNA user-provided sequences or an RNA sequence and up to 100 protein sequences provided by the user. Further, the server may also be probed to query RPIntDB (Muppirala et al., 2011), a database of known RNA-protein interactions, using a protein query sequence. Another method is PS-PRIP (Muppirala et al., 2016), a sequence motif-based method for “partner-specific” interfacial residue prediction. Here, short strings of amino acids or ribonucleotides that are composed of interacting residues are extracted as n-mer motifs from a dataset of 1,408 protein-RNA complex structures from the Protein Data Bank (PDB). These are stored in a look-up table that is scanned to identify such motifs in query protein or RNA sequences.

Many methods have been developed to predict residues involved in RNA interaction given an amino acid sequence (Figure 1, Supplementary Table S1). Of these, methods that focus on interface residue prediction use properties such as charge, amino acid composition, van der Waals volume, polarity, *etc.* These methods extract various features from amino acid residues and use them as the input to train the machine-learning models for classification. Several algorithms like SVM (Cai et al., 2003; Kumar et al., 2008; Murakami et al., 2010; Wang et al., 2010; Walia et al., 2014; Yang et al., 2015a; Bressin et al., 2019; Su et al., 2019; Qiu et al., 2020), neural networks (Alipanahi et al., 2015; Peng et al., 2017; Yan and Kurgan, 2017; Deng et al., 2018; Zhao and Du, 2020; Zhang et al., 2021b; Sun et al., 2021; Li and Liu, 2022; Zhang et al., 2022), naive bayes classifier (Sharan et al., 2017; Deng et al., 2021; Li et al., 2022) etc, as listed in Supplementary Table S1, have been successfully implemented. A common limitation faced by such ML-based methods is that the extracted features may be poorly representative of the physicochemical and environmental properties of amino acid residues, or their simplistic combination may introduce redundancy and affect overall prediction power of the approaches.

Structure-based methods to predict such residues are also popular and have benefitted from the availability of detailed structural information for more than 1,000 RNA-protein complexes in the PDB (Berman et al., 2000). In parallel, methods that address the partner prediction problem are also available (Muppirala et al., 2011). Such approaches derive from limited information on RNA-protein interaction partners in primary resources such as the PDB (Berman et al., 2000) and nucleic acid database/ NDB (Coimbatore Narayanan et al., 2014), and secondary resources such as PRIDB (Protein-RNA interface database) (Lewis et al., 2011) and BIPA (Biological Interaction database for Protein-nucleic Acid) (Lee and Blundell, 2009). They also use experimental data from *in vivo* or *in vitro* cross-linking studies that are focused on individual proteins or high-throughput RNA-binding microarray data, stored in repositories such as NPInter (noncoding RNAs and protein related biomacromolecules interaction database) (Wu et al., 2006; Teng et al., 2020), CLIPZ (database of post-transcriptional regulatory elements (RNA-binding proteins) built from cross-linking and immunoprecipitation data) (Khorshid et al., 2011) and RBPDB (database of RNA-binding protein specificities) (Cook et al., 2011). Other methods such as RBRIdent (identification of RNA-binding residues) (Xiong et al., 2015) use a genetic algorithm and integrate sequence and structure features by statistical analysis of interaction preferences between amino acid residues and their RNA partners from structure databases.

Tools like RBind (Binding sites on RNA) (Wang et al., 2018; Wang and Zhao, 2020), NAPS (network analysis of protein structures) (Chakrabarty et al., 2019) and PRIdictor (Protein-RNA Interaction predictor) (Tuvshinjargal et al., 2016) use a structural network approach for predicting such interactions. These methods tackle the challenge to predict not only RNA binding sites in proteins but also protein-binding sites in RNA. Several servers such as omiXcore (Armaos et al., 2017) use available CLIP data to predict amino acid residues. Here, a non-linear algorithm is trained on pooled RNA-protein interactions and accepts the proteins and large RNAs with a size between 500 and 20,000 as inputs. PRIP (protein-RNA interface predictor) uses a novel sequence semantics-based method to predict RPI (Li et al., 2022). Integrated classical machine and deep learning classifiers in methods such as RPI-MDLStack (RNA–protein interactions through deep learning with stacking strategy and LASSO) (Yu et al., 2022) have shown improved and robust performance in predicting such interactions. The accuracies of these programs range from 0.75 to 0.98.

### 2.2 Methods that predict protein-RNA interactions using structure information

Protein-RNA interactions can be predicted using structure-based information. These methods became possible as the number of protein-RNA complex structures deposited in the PDB increased in numbers. Here, either the availability of structures or structure predictions of protein, RNA or both as a complex have been employed to develop several approaches.

#### 2.2.1 Protein structure as input

16% of the tools to predict RPI have been developed based on the structure of a protein (Figure 1). Initially, many combinations of either sequence-based or structure-based features were applied to obtain predictions of protein-RNA interaction residues. These included physicochemical features, side-chain environment, sequence conservation score, position-specific scoring matrices (PSSMs), relative accessible surface area (RASA), secondary structure (SS), interaction propensity and so on. PredRBR (Liu et al., 2017) is one of the noteworthy approaches that employs 189 features which are extracted from sequence, structural and energetic characteristics, as also two categories of Euclidian and Voronoi neighborhood features that are derived from protein-RNA complexes in the RBP170 dataset compiled by Lewis et al., 2010 (Lewis et al., 2011). It employs an mRMR-IFS (maximal relevance minimal redundancy) approach to select an optimal subset of 177 optimal features and a gradient tree boosting algorithm for regression and classification to derive a model, that is useful to predict such interaction sites in datasets that were not employed in the training of the algorithm. The use of such approaches was found to reduce the computational time and improve the performance overall. The results also highlight the benefits of basing RNA-binding residue prediction method on the Gradient tree boosting (GTB) algorithm and structural neighborhood characteristics (Euclidian and Voronoi).

Another popular method is PRNA (Liu et al., 2010) which employs a random forest method for predicting RNA binding sites in proteins, using features that have been extracted from representative protein-RNA complex structures derived from RsiteDB (Shulman-Peleg et al., 2009) (listed in Supplementary Table S1). The strength of the method is that these features are a combination of sequence and structure features. The authors have developed a method by considering the neighborhood of amino acids in the interaction sites since amino acids with different neighborhoods or in different local structures often exhibit preferences for their RNA partners. A combination of interaction propensity feature between the amino acid and its interacting nucleotides that considers neighborhood and individual residue properties defined by six descriptors including physicochemical characteristics, hydrophobic index, relative accessible surface area, secondary structure, sequence conservation score and side-chain environment was found to be a powerful combination and resulted in high accuracy in prediction of known and annotated protein-RNA interaction sites. Another method by Go and co-workers examines amino acid singlet and doublet residue propensity at known protein-RNA interfaces obtained from the (protein quaternary structure) PQS server (Kim et al., 2006) and PSSMs from homologous sequences to make predictions. Such approaches aim to capture not only the pairing preference of amino acid types through propensity calculations but also shed light on the co-operative contribution of various interactions that are known to lie at such interfaces (Kim et al., 2006). Trends such as the high preference for Arg, Lys or aromatic residues such as Tyr to occur at the interface and favoring interactions with RNA were gleaned through these studies. Prediction accuracy of these methods, while reasonably sensitive, would benefit extensively with the inclusion of more structures of such complexes as and when solved, since their analysis is primarily statistical in nature. Such methods can be applied to predict protein–RNA interface residues for query protein structures without biochemical or functional data. Other approaches include PST-PRNA (Li and Liu, 2022) that employs protein surface topography (PST), physicochemical characteristics, structural information, PSSM features and a deep residual network approach, SNB-PSSM (Liu et al., 2021a) that uses a spatial neighbor–based PSSM for extraction of evolutionary information and an SVM as a classifier, NABind (Sun et al., 2016) that includes novel features such as residue electrostatic surface potential and triplet interface propensity in a random forest algorithm. Other ML-based methods include RBscore and NBench (Miao and Westhof, 2016), RBRDetector (Yang et al., 2014), DR_bind1 (Chen et al., 2014), aPRBind (Liu et al., 2021b) and aaRNA (Li et al., 2014).

#### 2.2.2 RNA structure as input

The shape and geometry of RNA can significantly influence RBP binding. Zhang and co-workers developed DeepNet-RBP (Zhang et al., 2015) which uses a deep learning framework to integrate RNA sequence, secondary and tertiary structural profiles, and constructs a ified representation to extract hidden structural features of RBP targets. The three main phases in their development include a data encoding phase of RNA sequences from CLIP-based experiments that were subjected to secondary structure prediction. Likewise, probable tertiary structural motifs were also derived for the sequences and encoded. In the training phase, a multimodal deep belief network (DBN) was employed to integrate the encoded sequence and structural profiles for available CLIP-seq datasets. The primary sequence and secondary structure automatically extract effective hidden structural features from the encoded raw sequence and structural profiles to predict RPI. Applications of this method were shown to effectively detect novel RBP binding sites on genomes and predict RBP binding sites in polypyrimidine tract-binding protein (PTB) and in internal ribosome entry site (IRES) segments and achieved an accuracy of 0.8–0.9. RBind and RBindS (Wang et al., 2018; Wang and Zhao, 2020) uses a network approach and RPI-net (Yan et al., 2021) which makes use of graph neural networks, are other powerful methods. NABS (Jiang et al., 2022) uses an integrative framework with both machine learning and template-based classifiers to predict binding nucleotides.

#### 2.2.3 Prediction using both RNA and protein structures as input

GraphProt (Maticzka et al., 2014) is a flexible machine learning framework that is capable of deriving learning models of RBP binding preferences from high throughput experimental data such as CLIP-seq and RNAcompete (Ray et al., 2009). Here, RNAshapes (Steffen et al., 2006) tool is employed to predict the secondary structure of the bound RNA. This input is used to encode the bound RNA sequence and structure in a graph that preserves base-pairing information, which is fed into a support vector machine to classify RBP bound sites from bound sites using efficient graph kernels (Maticzka et al., 2014). When applied, both sequence and structure logos can be derived for an input query or to predict novel RBP sites. GraphProt can detect the binding sequence and structure preference of RBPs and further predict the RBP binding sites on any input RNAs. The machine leaning-based methods are trained in an RBP-specific manner since RBPs have different binding preferences. One of the main applications of this approach is the computational ability to detect targets of an RBP. Indeed, a user case scenario presented by the authors derives a model based on CLIP-seq data from kidney cells to identify potential targets in the entire transcriptome. Further, if affinity data from RNAcompete experiments are available, GraphProt can apply a regression approach that can distinguish target sites according to their binding strength as well. DLPRB (Ben-Bassat et al., 2018) uses both CNN and recurrent neural network (RNN) to predict protein-RNA interactions and could achieve area der curve (AUC) of 0.81. RCK (Orenstein et al., 2016) is an extension of RNAcontext by a k-mer sequence- and structure-based binding model (Luo et al., 2017). RPI-BIND (Luo et al., 2017) analyses solved RNA-protein structural complexes and employs protein local conformations (PLCs) and 12 classes of RNA local conformations (RLCs), to train a model and predict such interactions. PRIME-3D2D (Xie et al., 2020) is another tool that can predict protein-RNA complex structure and is amenable to genome-scale binding site prediction of proteins on RNA. It uses an alignment-based approach involving TM-align (Zhang and Skolnick, 2005) and LocARNA (multiple alignment of RNA) (Will et al., 2007), to model the protein-RNA complex from which interactions are inferred.

### 2.3 Tools for modelling or docking of Protein-RNA complexes

The availability of protein-RNA complex structure is important for an derstanding of the function of the complex at a molecular level. As of 2022, 4300 macromolecular complexes containing both protein and RNA (excluding RNA/DNA hybrids) were available in the Protein Data Bank (PDB) (Berman et al., 2000). However, due to the inherent limitations of experimental techniques, this is only a small fraction of all the structures deposited in PDB and all identified protein-RNA interactions. Computational modelling of protein-RNA complexes is one of the approaches by which complex structure can be predicted and employed further to study intermolecular interactions. Nithin and coworkers provide a detailed review on available bioinformatic tools for protein-RNA docking (Nithin et al., 2018). As in the modelling of protein-protein interactions, protein-RNA complexes also involve structural and physicochemical complementarity and have borrowed from existing protein-protein docking methods such as HDock, GRAMM, ZDock, RosettaDock *etc.* (Tovchigrechko and Vakser, 2006; Guilhot-Gaudeffroy et al., 2014; Pierce et al., 2014; Yan et al., 2017). Both template-based modelling and free docking are employed to predict binding modes of interactions in protein-RNA complexes. A recent study has highlighted some of the available tools for modelling such complexes (Madan et al., 2016). PRIME (Zheng et al., 2016) is a template-based comparative modelling program for protein-RNA interaction modelling in which TMalign (protein structural alignment) and RMalign (RNA structural alignment) are both employed for identifying a template for the protein and RNA sequence of interest. The output of the program is the modelled protein RNA-complex structure which is ranked based on a score like TMscore. A limitation faced by this approach is the inability to build a model in the absence of a reliable template. Further, the scoring function of the RNA alignment algorithm in PRIME is size-dependent, which limits its ability to detect good templates in some cases. The authors report that, like protein-protein complexes, the correlation of protein-RNA structural similarity with the binding mode is poorer on account of greater RNA flexibility. RStrucfam also offers three-dimensional models, once there is an association with a family of RBP (Ghosh et al., 2016).

An approach to model protein-RNA complexes in the absence of a structural template of the complex is to model the protein and RNA components individually and then perform docking and is referred to as free docking, as implemented in 3dRPC (Huang et al., 2018). Here, the docking method RPDOCK, a protein-RNA rigid docking protocol considers geometric and electrostatic factors such as atom packing, residue preferences, stacking interactions between bases and aromatic residues in generating the decoy poses that are evaluated using a specific scoring function. Despite these strengths, the inherent flexibility of RNA and the selection of the appropriate model from the large number of generated docking poses can present a computational challenge for the approach (Huang et al., 2013; Xie et al., 2020). One of the most cited methods for protein- RNA docking is HDOCK (Kim et al., 2014) which employs a hybrid docking algorithm of template-based modelling and free docking based on iterative knowledge-based scoring function. Here, both protein sequence and structure are accepted as inputs while for RNA only structure input is accepted due to inherent challenges in modelling RNA. If binding site information is available, this may be submitted prior to the analysis. A sequence similarity search is performed to find an ideal template from the PDB database using HHsearch (Fidler et al., 2016) for the protein sequence while FASTA is employed to find homologues for the RNA sequence. If the template identified is the same, the model for protein and RNA are derived using the template complex structure, else they are built individually based on different templates using Modeller (Šali and Blundell, 1993). A hierarchical Fast Fourier transform (FFT)-based docking program, HDOCKlite is then employed to sample binding orientations between the two models that are scored using a shape-based pairwise scoring function and over 100 binding models are generated for the user. The method may face limitations when the search algorithm is able to find a suitable homolog. Details on popular methods employed in docking are listed in Supplementary Table S1.

One of the challenges faced by rigid-docking methods involving RNA is the requirement for a suitable template. Several methods have become available to predict RNA 3D structure over the past few decades (Supplementary Table S1). Like proteins, 3D structure of RNA is also conserved and predictable. Therefore, alignment of RNA with previously determined RNA structures will help to predict their 3D structures and identify motifs that are significant for functions like ligand binding and active site. A popular method is EvoClustRNA (Magnus et al., 2019) which applies a multi-step modelling process and considers that RNA sequences from the same RNA family fold into similar and conserved structures. Such homologs are identified through searches in the Rfam database (Kalvari et al., 2021). Independent folding simulations are then performed, and the model selection is based on the most common structural arrangement of the common helical fragments. Current RNA folding algorithms can predict RNA structures of short to very long RNA sequences. Structure-based prediction algorithms (Das and Baker, 2007; Das et al., 2008; Parisien and Major, 2008; Das et al., 2010; Cao and Chen, 2011; Zhao et al., 2012; Magnus et al., 2019; Andrikos et al., 2022) achieve the highest accuracy with efficient alignments. A part of a well-aligned structure can be extracted and used as a fragment and many such fragments can be assembled on a template to form a 3D structure. Indeed, such methods aim to overcome limitations of template-based modelling which do not consider the flexibility of the RNA molecules, and the number of known RNA structures. *Ab-initio* tools (Das and Baker, 2007; Jossinet et al., 2010; Magnus et al., 2014; Xu et al., 2014; Mallet et al., 2022), on the other hand, face challenges such as the length of RNA, conformational sampling, evaluation of energies for the tertiary contacts, and knowledge-based energy functions. Coarse-grained approaches (Wang et al., 1999; Cao and Chen, 2005; Tan et al., 2006; Ding et al., 2008; Sharma et al., 2008; Jonikas et al., 2009; Pasquali and Derreumaux, 2010; Xia et al., 2010; Izzo et al., 2011; Xia et al., 2013; Kim et al., 2014; Shi et al., 2014; Oliver et al., 2022) model entire RNA structure using beads and these models are subjected to energy minimization, molecular dynamics and/or Monte Carlo simulations. These methods offer a way to derstand RNA folding and show improved efficiency in prediction of lengthy RNA molecules. Coarse-grained models, however, face limitations in entropy calculations and long-range tertiary interactions.

Future improvements in methods to model Protein-RNA complexes could focus on combinations of free docking and template-based algorithms because using either approach independently has a reduced accuracy of prediction.

### 2.4 Tools for studying and analysing protein-RNA interfaces

Evaluation of phenotypic impact of sequence variations is very important to derstand function. There are various tools for analysing the effect of mutations in protein-RNA complexes. ENTANGLE (Allers and Shamoo, 2001) is a structure-based analysis program, to analyze protein-RNA interactions that allows users to examine protein-RNA interactions in an available three-dimensional structure or model. Users can choose from a range of ways to study protein-RNA interactions and can examine the interactions through a graphics interface. In addition, the tool has been employed to build a protein-RNA interaction database that catalogs the various types of characteristic interactions of these complexes and considers electrostatic, hydrogen bonding and stacking interactions. Such studies and databases have provided useful insights to distinguish protein-RNA binding from other protein-DNA binding. mCSM-NA (Pires et al., 2017), DeepCLIP (Bjørnholt Grønning et al., 2020) and PremPRI (Zhang et al., 2020) are a few examples of tools which use machine learning to calculate binding affinity changes in a protein complex upon mutation. mCSM-NA employs graph-based signatures to represent protein-nucleic acid complexes and models the distance patterns in wild type proteins from the ProNIT database (Prabakaran et al., 2000). Residues in the vicinity of the mutated protein are labelled based on pharmacophore modelling which describes the geometry and physicochemical properties of the residue environment. A machine learning based model is then trained on the effects of such mutations to arrive at models that can predict changes in nucleic acid binding affinities. Input for the server is a protein-RNA complex structure and up to a maximum list of 20 single point mutations. The output includes predicted changes in binding affinity in Kcal/mol, the relative residue solvent accessibility of the mutated residue, predicted change in binding affinity and protein stability. Limited success of these techniques can be attributed to the intramolecular interactions and various conformations that are not considered in the physical models. Accurate prediction of RNA–protein binding affinities is very challenging, and a complete prediction framework for RNA–protein complexes is yet to be developed. Supplementary Table S1 lists and highlights features of various tools that are available to study interactions in these interfaces.

### 2.5 Tools for analysing hotspots in protein-RNA complexes

Conserved residues in binding sites that contribute to the strength of binding, and whose substitution to Alanine leads to an increase in the binding free energy (ΔG) of at least 2.0 kcal mol─1, are defined as hotspot residues (Clackson and Wells, 1995). Given the complex structure, many tools are available to predict hotspots in protein-RNA interfaces and predict the quantitative changes in free energy or a probability score for a hotspot residue. All these tools are built on thermodynamics data. PrabHot (Krüger et al., 2018), XGBPRH (Deng et al., 2019) and SREPRHot (Zhou et al., 2022) use ML algorithms and structural information to predict hotspot residues. Input for PrabHot, a webserver, is a protein-RNA complex structure. The method is based on 47 protein-RNA complexes from which various features such as network, exposure, sequence and structure determinants are extracted. An ensemble approach is employed to integrate SVM (Support Vector Machine), GTB (Gradient Tree Boosting) and ERT (Extremely Randomized Trees) based classification to predict the effect of mutations. Other methods include SPHot (Zhang et al., 2019) and iPNHOT (Zhu et al., 2020) that are based on SVM analyses of hotspot residues from protein sequences (Supplementary Table S1). SPHot uses only sequence information for the prediction. Although many tools are available, this area still has many limitations and challenges. These include lack of thermodynamic data, experimentally verified data sets and structural data. In future, we can expect that these tools can be improved to include the multiple conformational nature of protein-RNA complexes.

### 2.6 Tools for studying the structural dynamics of protein-RNA complexes

Functions of protein-RNA complexes are intimately linked to their dynamics. Long-timescale molecular dynamics (MD) simulations have been successfully used to characterize complex conformational transitions in proteins. In principle, MD simulation is a powerful tool for characterizing such conformational changes in RNA molecules, which depends on force-field parameters. AMBER force field (Tan et al., 2018) uses a combination of *ab initio* and empirical methods, modified electrostatic, van der Waals (vdW), and torsional parameters to accurately reproduce the energetics of nucleobase stacking, base pairing, and key torsional conformers. CHARMM modified its forcefield parameters to make it suitable for nucleic acids (Xu et al., 2016). OPLS-AA force field made changes in potential energy surfaces of the backbone α- and γ dihedral angles, for modelling of RNA (Robertson et al., 2019). Apart from this there are advanced quantum mechanics/molecular mechanics (QM/MM) computations which could be used to indirectly rationalize problems seen in MM-based MD simulations of protein–RNA complexes (Pokorná et al., 2018). Martini force field (Monticelli et al., 2008) helps in coarse-grained representation of protein-RNA complexes, and these models can be applied in MD simulations. Elastic network representation of protein-RNA (Pinamonti et al., 2015) complex can be employed in normal mode analysis which can be used to track flexibility and dynamics of a complex.

### 2.7 Databases for protein-RNA interactions

Various databases provide comprehensive repositories of RNA-protein interaction data that have been gathered from experiments, literature and other databases and computational predictions. PRD (Fujimori et al., 2012), NPInter (Wu et al., 2006; Teng et al., 2020), RNAct (Lang et al., 2019) and RAID (Zhang et al., 2014) help us integrate data from various platforms. CLIPdb (Yang et al., 2015b) and RPI- PRED RNA (Suresh et al., 2015) curate experimental information from literature and capture this in a table. PRIDB (Lewis et al., 2011) and RBPDB (Cook et al., 2011) characterize and provide data with the help of existing structural information. Protein family databases such as PFAM also contain RNA binding domain families. Nearly 3909 domain families are associated with the keyword RNA-binding. Such domain families that are represented as Hidden Markov models (HMM) are useful in sequence-based association of RNA binding function in a query protein sequence, using HMMPFAM (SR Eddy, 2004). There are also specialized databases, such as EcRBPOME (Ghosh et al., 2019) which offer putative RNA binding proteins in a genome-wide scale in around 600 *E. coli* strains. Among the above databases, NPInter, PRD, RNAct, CLIPdb and RBPDB provide and extract information from experimental techniques. In future, an exhaustive interaction database which could store detailed and multi-dimensional information about an interaction entry, such as a binding region/motif, structure detection method, interactions, *etc.* would add more value to such efforts. Furthermore, the ability to predict potential RPI is based on availability of such catalogued information, making such databases necessary and valuable.

## 3 Conclusion

This review provides a comprehensive survey of computational tools for the analysis of protein-RNA interactions. The availability of mathematical models and profiles, together with sensitive search algorithms, enable effective association of new genes into pre-existing families of RNA-binding proteins from mere sequence information. Repositories such as protein sequence families (PFAM) (Finn et al., 2016; Mistry et al., 2021) and in-built databases in servers such as RStrucFam (Ghosh et al., 2016) make focused searches easier. As seen in the pie-chart (Figure 1), majority of the tools that have been developed predict amino acid residues and very few tools are available for modelling protein-RNA interactions. Nearly 30% of the tools are for predicting interactions in the protein-RNA complexes given a protein query sequence. There is still room for better methods and approaches to evolve. In this section, we discuss some of the inherent challenges and outstanding issues.

RBPs are modular and may possess more than one RBD that are connected with flexible linkers or may occur as isoforms. Automatic tools are not available to provide accurate assignment of domain boundaries and nomenclature. Which of these domains bear sequence signatures of functionally important residues, how they co-operate in exhibiting function and their relative affinity for RNA still are open questions. Several proteins that contain RNA-binding domains also contain substantial stretches of disordered regions. Their structural and functional regulation are hard to capture and remain a treasure-house of knowns. Despite the choice of objective mathematical models, presence of spatial motifs of RNA-binding residues or RNA that acquire three-dimensional structure might escape detection. Some RBPs are known to bind both DNA and RNA and this might cause sufficient confusion in prediction since the results may be viewed as false positives.

The success of computational algorithms is usually measured using statistical parameters such as accuracy, sensitivity and specificity. The performance of algorithms to recognize RNA-binding proteins is challenged by the absence of a comprehensive and reliable gold-standard to look upon. It is now accepted that different databases of RNA-binding proteins that use different high-throughput experimental approaches and platforms do not agree very well with each other (Ghosh and Sowdhamini, 2016). It would be desirable to arrive at an ified experimental approach for the identification of RNA-binding proteins.

Finally, RNA-binding proteins play important roles in diverse biological roles such as developmental processes and ageing. They are also amenable to functional regulation such as phosphorylation switches and expression levels. In future, meta-analyses are required to assimilate such functional information.

## Glossary

ncRNA: non-coding RNA
miRNA: microRNA
piRNA: Piwi-interacting RNAl
ChIP-seq: Chip sequencing
HT-SELEX: High throughput systematic evolution of ligands by exponential enrichment
PPrint: Prediction of Protein RNA-Interaction
CLIP: cross-linking immunoprecipitation
RBPmap: mapping binding sites of RNA-binding proteins
RNApin: Protein Interacting Nucleotides (PINs) in RNA sequences
EDCNN: Evolutionary Deep Convolutional Neural Network
catRAPIDomics: computation of protein–RNA interaction propensities at the transcriptome-and RNA-binding proteome
RPISeq: RNA-Protein Interaction Prediction
RPIntDB: database of known RNA-protein interactions
PS-PRIP: partner specific protein RNA interaction prediction
PredRBR: predict RNA-binding residues
mRMR-IFS: Minimum Redundancy Maximal Relevance (mRMR) method followed by incremental feature selection (IFS)
PRNA: Promoter-associated RNAs
RsiteDB: RNA binding sites database
PST-PRNA: predicting RNA-binding sites (PRNA) based on protein surface topography (PST)
RBscore: RNA-/DNA-binding residues in proteins and visualizes the prediction scores
NBench: Name of a benchmark database
RBRDetector: RNA-Binding Residue Detector
DR_bind1: a web server for predicting DNA-binding residues from the protein structure based on electrostatics, evolution and geometry
aPRBind: *ab initio* algorithm *ab initio* Protein–RNA Binding site prediction
DeepNet-RBP: An algorithm using Deep belief network (DBN) to predict RBP binding sites
aaRNA: aaRNA is an RNA binding site predictor
RBind: A computational network method to predict RNA binding sites
RBindS: A user-friendly server for RNA binding site prediction
RPI-net: RNA Protein Interaction prediction using Neural Networks
NABS: An algorithm for Nucleic Acid Binding Site prediction
GraphProt: a flexible machine-learning framework for learning models of RBP binding preferences
RNAcompete: RNA compete is an *in vitro* protein-centric method used to analyze RNA-protein interactions
RNAshapes: an integrated RNA analysis package based on abstract shapes
DLPRB: A Deep Learning Approach for Predicting Protein-RNA Binding
RPI-BIND: a structure-based method for accurate identification of RNA-protein binding sites
HDock: Protein-protein and protein-DNA/RNA docking based on a hybrid algorithm of template-based modeling and *ab initio* free docking
HDOCKlite: Another version of HDock
GRAMM: The Global RAnge Molecular Matching
ZDock: An initial-stage protein-docking algorithm
RosettaDock: Docking algorithm developed as a part of Rosetta platform
PRIME: Protein-RNA Interaction ModElling program
RStrucfam: a web server to associate structure and cognate RNA for RNA-binding proteins from sequence information
3dRPC: 3-D RNA_protein structure prediction
HHsearch: profile‐profile tool that explicitly weights a proportion of an alignment to aligned (predicted) secondary structure. MSAs can be built by either PSI‐BLAST or HHblits
EvoClustRNA: A clustering routine of evolutionary conserved regions (helical regions) for RNA fold prediction
Rfam: an RNA family database
ENTANGLE: a JAVA program that classifies and sorts potential protein-nucleic acid interactions
mCSM-NA: mutation Cutoff Scanning Matrix applied to Nucleic Acids
DeepCLIP: predicting the effect of mutations on protein–RNA binding with deep learning
PremPRI: Predicting the Effects of Missense Mutations on Protein-RNA Interactions
ProNIT: A database for protein nucleic acid interactions
PrabHot: Prediction of Protein-RNA Binding Energy Hot Spots
XGBPRH: an eXtreme Gradient Boosting (XGBoost) algorithm for Protein-RNA Hotspot prediction
SREPRHot: a SMOTE and Random grouping strategies-based Ensemble learning model for Protein-RNA binding Hotspot prediction
SPHot: Sequence-based Prediction of RNA-binding Hot Spots
iPNHOT: identifying protein-nucleic acid interaction hot spots
AMBER: Assisted Model Building with Energy Refinement
CHARMM: Chemistry at Harvard Macromolecular Mechanics
PRD: A protein-RNA interaction database
NPInter: the noncoding RNAs and protein related biomacromolecules interaction database
RNAct: protein–RNA interactome
RAID: RNA-associated (RNA–RNA/RNA–protein) interaction Database
CLIPdb: a CLIP-seq database for protein-RNA interactions
RPI: PRED RNA—Another version of RNA-protein interaction prediction algorithm
PRIDB: a protein—RNA interface database
RBPDB: RNA-Binding Protein DataBase
